# Shiny Science: A New Substitute for Hexavalent Chromium

**DOI:** 10.1289/ehp.114-a482

**Published:** 2006-08

**Authors:** Lance Frazer

Although perhaps more familiar to those of us of a certain age who remember when all cars had sparkling mirror-finish bumpers, chromium still plays a big part in industry. Chromium is valued for its brightness, durability, resistance to corrosion, and hardness. It is used as a pigment in paint, inks, and plastics, as an anticorrosion agent in protective coatings, and in chrome plating on such things as aircraft engine components, tool and die parts, railroad wheel bearings, and, of course, the “brightwork” that trims motorcycles, cars, and trucks. As more and more scientific studies have revealed, however, chromium also has a darker side. The chromium used in the plating industry is primarily hexavalent chromium, which is a very different animal from the trivalent form required by the human body. Hexavalent chromium is a potent human carcinogen, and can also cause dermal irritation and kidney and liver damage. Now, in an effort to find safer alternatives, researchers are looking at tailored nanostructures that offer the appearance and durability of hexavalent chromium without the hazards.

## How Electroplating Works

Electroplating involves immersing the metal parts to be plated in a bath of chromium trioxide (CrO_3_), typically prepared by dissolving crystalline CrO_3_ in a mix of distilled water and sulfuric acid. A direct current is passed through the solution, and the resulting reaction leaves a deposit of chromium on the piece being plated.

One problem in this process is the production of hydrogen and oxygen at the electrodes. The gas bubbles to the surface, creating a mist of the plating solution (which contains hexavalent chromium) that must be controlled. Additionally, mechanical agitation of the bath (used to improve plating quality) can also result in the release of this hazardous mist.

According to Steve Smith, a supervising industrial hygienist with the California Occupational Safety and Health Administration, the permissible exposure limit (PEL) for workers in the chrome-plating industry is set for airborne concentrations based on the average over an eight-hour workday. In February 2006, the federal PEL for hexavalent chromium was reduced from 52 μg/m^3^ to 5 μg/m^3^. Although the federal government mandates PELs, states have the individual authority to regulate substances of concern more strictly.

Smith says different chromium compounds are regulated to a greater or lesser extent than others, depending upon the other substances involved. Lead chromate, for example, contains not one but two substances of marked concern, and thus is regulated at lower exposures. Similarly, strontium chromate (used in paint) has a much lower PEL in California (0.5 μg/L^3^) than hexavalent chromium because of studies showing that it’s far more toxic than its chromium consitutent alone.

Health and industry officials are somewhat at odds over the level at which hexavalent chromium should and can reasonably be regulated. “I’m not an alarmist,” says Neal Langerman, principal scientist with the consultancy Advanced Chemical Safety. “On your car bumper, chromium is a very low-risk substance, but certainly the act of plating carries a much higher risk. Hexavalent chromium is a confirmed carcinogen, and ingestion or inhalation over a period of time can cause serious, ultimately fatal, impacts.” Smith says the recent decision to go to 5 μg/m^3^ represented the best possible solution to both health concerns and industry economic concerns.

## The Search for Alternatives

Industry has tried using other substances in place of hexavalent chromium to achieve the same results. Any alternative would need to duplicate the desired properties of the original chemical without requiring an extensive revamping of the entire plating process. Trivalent chromium is used to some extent, but the industry still has some concerns with color issues, which matters when a bright, reflective surface is desired. Further, unless extensive preparations are used, corrosion resistance is not as high as with hexavalent chromium. For some uses, the industry has begun experimenting with thermal spraying using a tungsten carbide substitute as an alternative to chrome baths. However, retooling a shop for this method can be expensive.

Other researchers are thinking smaller—much smaller. Christopher Schuh, an associate professor in the Department of Materials Science and Engineering at the Massachusetts Institute of Technology (MIT), and former MIT researcher Alan Lund are manipulating nickel and tungsten at the atomic level to create a more environmentally friendly alternative to hexavalent chromium. Working with them is Andrew Detor, a graduate student in the MIT Department of Materials Science and Engineering.

Schuh says his goal was to “address some of the shortcomings in our current suite of metals. There has been a lot of work in tailored nanostructures to develop new materials with new properties, and this seemed like an ideal application.” He and Lund formed the Medford, Massachusetts–based Xtalic Corporation to take the technology into the commercial arena.

Schuh points out that the chromium coating industry is a multibillion-dollar industry, and the problems associated with hexavalent chromium account for a significant percentage of the process cost. “We’ve developed the ability to control the structure of metals at the nanoscale level,” he says. “Metals are, in general, composed of many crystal grains, and our work has been centered around controlling the size of these grains, enabling us to create new metals that deliver the properties of chromium without chromium’s environmental baggage. . . . We looked at the suite of properties that make chromium valuable and used nanoscale manipulation to duplicate those properties without hexavalent chromium.”

A good deal of the information regarding Schuh’s process is confidential under the proprietary interests of the new company. However, he can say that the basic plating process is little different from the conventional chrome plating process: “It’s in the design of the alloy and its structure that the art becomes new.”

## Atomic Energy

Schuh explains that tungsten atoms are about 10–12% larger than nickel atoms. “Because the atoms are of different sizes, it’s harder to pack them efficiently in a crystal,” he says. “Adding tungsten promotes the formation of more and smaller grains; as you add more mismatch to the system, you promote the formation of intercrystalline regions. And by controlling the grain size, you can have a direct impact on the properties of hardness, abrasion resistance, and so on.”

Schuh says his new coating hasn’t yet been tested across the broad spectrum of chromium’s applications. But tests to date have been promising. “We have looked at several of chromium’s key properties—reflectivity, for example. Side by side, I can’t tell the difference,” he says. “We’ve also tested our coating for use in a marine environment, where chromium is valuable because it protects steel against the corrosive effects of saltwater. In a side-by-side test, our coating outlasted chromium by a factor of more than ten.”

Anytime a new process is substituted for something that has been in proven use for some time, there may be a few snags. According to Schuh, the chemicals traditionally used in chrome plating are relatively inexpensive (mainly because of volume), while “our chemicals, because of not being used in the same volume, are somewhat more expensive.” However, he thinks that will change as the new process is scaled up to a commercial level—something he expects within a year or two.

He adds that in other cost-related areas, the new process is already better or has the potential to be so—for example, by saving on power costs through greater efficiency, and on labor costs through less finish work in many applications. Schuh explains that it can be quite difficult to get uniform coverage with chromium, especially on parts of complex geometry. The new coating goes down much more evenly, which reduces the need for post-plating grinding, machining, and buffing.

Schuh points out that his team deliberately designed a process that would work as a drop-in replacement. “In developing a process like this, you waste many of the benefits if you make it overly complex, or something that requires extensive retooling or redesign of existing process lines,” he explains.

The new process is not without its own potential hazards, however. There is a good deal as yet unknown about the emerging science of nanotechnology and the possible interaction of nanoscale materials with the environment and with the human body. According to NIOSH, materials exhibit unique properties at the nanoscale that affect their physical, chemical, and biological behavior.

Nickel, too, has its own regulatory issues. “Nickel is a very potent sensitizer, and we’ve seen it can cause a very serious allergic response,” says Langerman. “Of course, it all depends upon the end use. If you’re using it for corrosion control on aircraft parts, for example, it’s not going to be an issue. But you’ll still need employee protection against exposure, and you’ll have to be concerned about any end user contact.”

Still, says Smith, while nickel definitely has its own regulatory concerns, it’s conceivably less hazardous than hexavalent chromium. “The general concept of substituting a less toxic product for a more toxic one is always one of the best methods of controlling employee exposure,” he says.

## Little Structures with Big Potential

Schuh and colleagues see their new technique as a springboard, not an end point. “What we’ve done is to develop a process to make and put down new coatings using highly tailored nanostructures, so I could easily imagine new coatings with different metals,” Schuh says. “For example, many people are working with cobalt-based coatings because of their applications in biological fields, so that’s a possibility, and my sense is that it would be every bit as easy to use cobalt as to use nickel. And there are many other metals that could be equally applicable.”

Kent Peaslee, a professor of metallurgical engineering at the University of Missouri in Rolla, says based on what he’s seen, “Schuh is applying a new technology to try and solve a problem that a lot of people have done research on over the years. Anything you can do to reduce or eliminate the need for these types of coatings is a plus because it not only solves the problem of the plating, but it also eliminates the problem of disposal of the spent plating solutions. While I haven’t seen evidence of success yet, this looks like a process with real potential.”

## Hexavalent Chromium Exposure

### A Regulation Under Attack?

According to OSHA, some 550,000 workers are exposed to hexavalent chromium on the job. Are these workers being protected as well as they could be? David Michaels, head of the Project on Scientific Knowledge and Public Policy at the George Washington University School of Health, and colleagues from George Washington University and the watchdog group Public Citizen claim the chromium industry mounted an active campaign to weaken proposed standards and knowingly kept critical data from OSHA during the comment phase of the hearings to set new standards. Their report appeared 23 February 2006 in the online journal *Environmental Health: A Global Access Science Source*.

The occupational permissible exposure limit (PEL) for hexavalent chromium had been set at 52 μg/m^3^ since the 1940s. In 1997 and 2002, OSHA was sued to lower the exposure level to 0.25 μg/m^3^, leading to a 2002 order by the U.S. Court of Appeals to issue a final standard by January 2006 (later extended to February 2006). “Faced with the threat of stronger regulation,” Michaels and colleagues wrote, “the chromium industry initiated an effort to challenge the scientific evidence supporting a more protective standard.”

Michaels claims a 1998 study commissioned by a group of chromium manufacturers known as the Industrial Health Foundation found a significantly elevated risk of lung cancer at exposures just over 1 μg/m^3^. The research, he says, was finished by 2002 but the sponsors did not provide the study to OSHA during the hearing period. Additionally, Michaels says, the industry’s epidemiologists claimed the study had to be presented as separate cohorts—which rendered each component statistically underpowered—because of different exposure measurement methods, “when the original proposal said specifically that [they had] the methodology to combine these cohorts. A post hoc analysis led to a reshuffling and change of results. That’s not considered an ethical approach.”

In their article, Michaels and colleagues suggested that studies funded by private sponsors that seek to influence public regulatory proceedings should be subject to the same access and reporting provisions as those applied to publicly funded science. Parties in regulatory proceedings should be required to disclose whether the studies were performed by researchers who had the right to present their findings without the sponsor’s consent or influence, and to certify that all relevant data have been submitted to the public record, whether published or not.

Kate McMahon-Lohrer, an attorney with Kelley Drye Collier Shannon (formerly Collier Shannon Scott) who represented the chromium industry during the regulatory hearing process, characterizes Michaels’s allegations as false and misleading. “The primary allegation is that the chromium industry hid data,” she says, “but OSHA did get the relevant study, which was actually supplied by Public Citizen, and OSHA stated in their final ruling that they had considered the study, and it didn’t change their risk assessment conclusions.”

McMahon-Lohrer was also quoted in the 23 February 2006 edition of *USA Today* as saying that “OSHA knew of the research, but wouldn’t have accepted it until it was published in a peer-reviewed journal.”

Michaels replies that “this is simply false. Regulatory agencies want to see all relevant data and know how to weigh submitted literature differently if it’s not peer-reviewed. Claiming that OSHA insists on a peer-review process is merely a convenient excuse for not submitting relevant data.”

OSHA would not comment on the decision-making rationale behind its final PEL of 5 μg/m^3^ beyond referring to the listing in the 28 February 2006 *Federal Register*, which states, “The PEL established by this rule reduces the significant risk posed to workers by occupational exposure to [hexavalent chromium] to the maximum extent that is technologically and economically feasible.”

While preferring to avoid what he calls “a politicized shouting match,” Neal Langerman, principal scientist with the consultancy Advanced Chemical Safety, says, “I do feel that all good, nonpolitical science indicates [the need for] much lower levels of exposure”—even below the 5 μg/m^3^ PEL—“and I also know that exposure control engineering becomes more expensive at lower levels, so the whole thing of setting exposure levels seems a money-driven issue.”–**Lance Frazer**

## Figures and Tables

**Figure:**
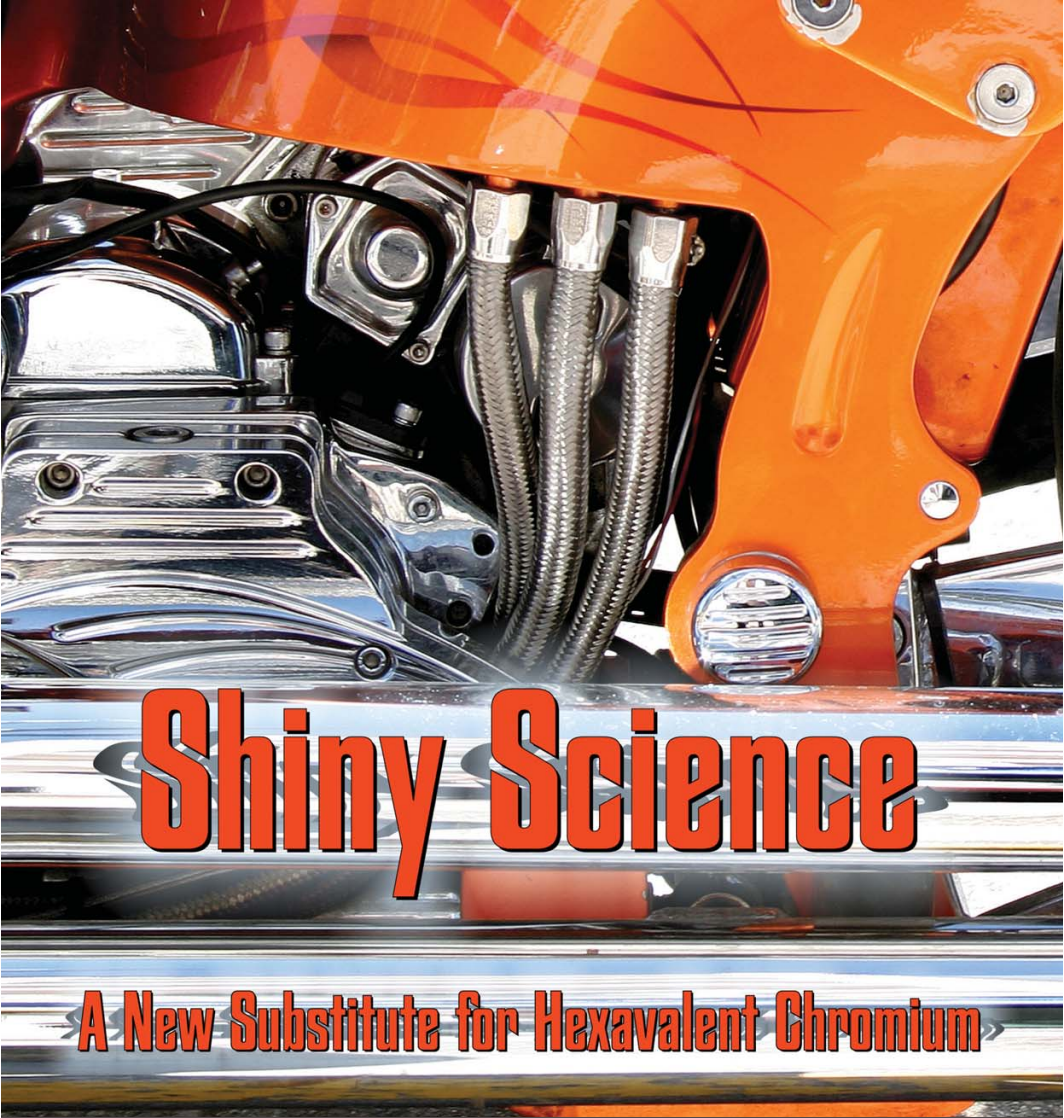


**Figure:**
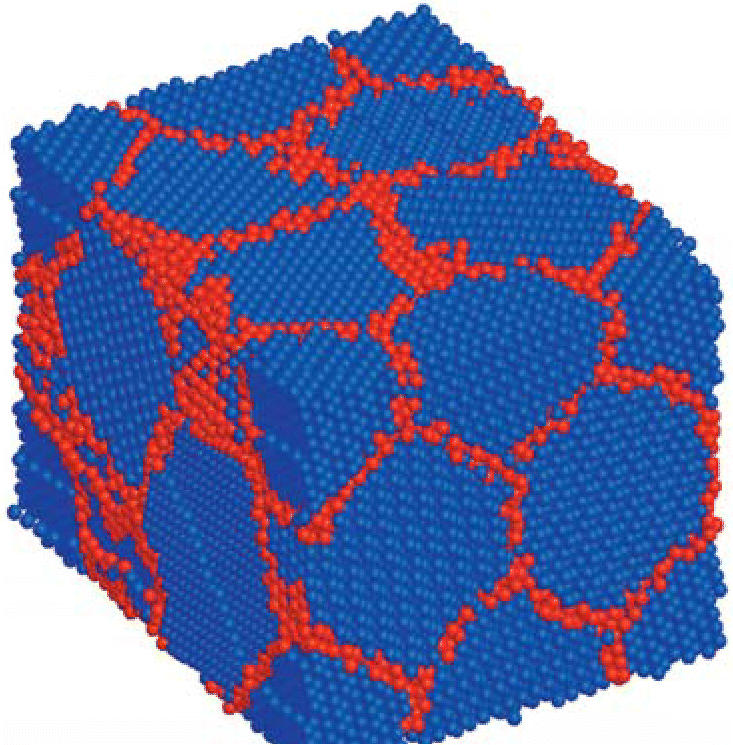
Metal by design A view of nickel–tungsten nanocrystalline alloy shows atoms within grains (blue) and at the grain boundary (red). Grain size helps determine hardness, abrasion, and resistance.
